# Hypertriglyceridaemia in extremely preterm infants receiving parenteral lipid emulsions

**DOI:** 10.1186/s12887-018-1325-2

**Published:** 2018-11-07

**Authors:** Ruth Sinclair, Tim Schindler, Kei Lui, Srinivas Bolisetty

**Affiliations:** 1 Royal Hospital for Women, Randwick, NSW Australia; 2 University of New South Wales, Randwick, NSW Australia

**Keywords:** Hypertriglyceridemia, Lipid emulsion, Preterm, Neonate

## Abstract

**Background:**

Lipid emulsions (LE) are routinely administered as part of parenteral nutrition in neonates. There is a wide variation in clinical practice of plasma triglyceride monitoring during LE therapy. Our aim was to evaluate the incidence of hypertriglyceridaemia (Plasma triglyceride > 2.8 mmol/L) and its association with mortality and major morbidities in extremely preterm infants on parenteral nutrition.

**Methods:**

A retrospective review of 195 infants < 29 weeks gestation. Lipid emulsion was commenced at 1 g/kg/day soon after birth and increased by 1 g/kg daily up to 3 g/kg/day and continued until the infant was on at least 120 ml/kg/day of enteral feeds. Plasma triglyceride concentrations were measured at each increment and the lipid emulsion dosage was adjusted to keep plasma triglyceride concentrations ≤2.8 mmol/L.

**Results:**

Hypertriglyceridemia was noted in 38 neonates (32.5% in 23–25 weeks and 16.1% in 26–28 weeks). Severe hypertriglyceridemia (> 4.5 mmol/L) was noted in 11 infants (10.0% in 23–25 weeks and 4.5% in 26–28 weeks). Hypertriglyceridemia was associated with an increase in mortality (unadjusted OR 3.5; 95% CI 1.13–10.76; 0.033) and severe retinopathy of prematurity (unadjusted OR 4.06; 95% CI 1.73–9.59; 0.002) on univariate analysis. However, this association became non-significant in multivariate analysis with adjustment for gestation and birthweight.

**Conclusions:**

Hypertriglyceridemia is common in extremely preterm infants receiving parenteral lipid emulsions. Regular monitoring and prompt adjustment of lipid intake in the presence of hypertriglyceridemia, minimising the length of exposure to hypertriglyceridemia, may mitigate potential consequences.

## Background

Lipid emulsions (LE) are a vital component of parenteral nutrition (PN) in preterm infants providing a low-volume source of essential fatty acids and energy [1, 2]. The timing and rate of commencement LE has long been debated. The European Society for Paediatric Gastroenterology Hepatology and Nutrition (ESPGHAN) 2005 guideline recommended that LE could be commenced on day 1, but no later than day 3 in order to avoid essential fatty acid deficiency [1]. A number of systematic reviews have shown no increase in side effects with early commencement of LE [2–4]. Potential benefits include improved nitrogen balance, anabolism and growth [4], better control of hyperglycaemia and reduced rates of retinopathy of prematurity (ROP) and necrotizing enterocolitis (NEC) [5].

Plasma triglyceride (TG) concentrations were performed as a safety variable in a number of clinical trials evaluating LE in neonates [6–11]. TG thresholds varied in these trials and adverse clinical outcomes were not reported in cases of hypertriglyceridemia (HT). Lack of reporting and the variation in TG thresholds for LE titration make it difficult to guide clinical practice in neonatal intensive care units (NICU). Adamkin et al. [12] suggested that preterm infants can tolerate serum triglyceride levels of up to 2.8 mmol/L (250 mg/dl) without any undesirable consequences. This was based on a study of only 10 infants with birth weights 1424 g ± 177 (SEM) started on 0.4 g/kg/day of LE increased up to 1.6 g/kg/day in the first week of life. ESPGHAN 2005 Guidelines recommend monitoring of triglycerides in preterm and term infants and suggest a triglyceride level of 2.8 mmol/L as the upper limit [1].

In Australasia, a consensus group of tertiary NICUs in the region has been standardizing newborn PN formulations culminating in an evidence based consensus statement in 2012 [13]. One of the recommendations was the timing of commencement of parenteral LE would be determined by individual NICUs with day 1 administration as an option (LOE 1, GOR C). No consensus could be reached on the need and/or frequency of plasma triglyceride (TG) measurements. In February 2015, as part of the ANZNN Parenteral Nutrition Network Meeting, a survey of participating NICUs in the group found that 98% commenced LE within 2 h of birth in extremely preterm infants but only 8% routinely monitored plasma TG concentrations (unpublished).

Our NICU introduced the consensus group guidelines and PN formulations in July 2012. As part of the new guidelines, we introduced routine titration of LE infusion according to plasma TG concentrations. The objectives of this quality improvement study were to measure compliance to the unit policy, report the incidence of HT and evaluate any association between HT and neonatal mortality and morbidity.

## Methods

This was a retrospective case note review of all infants less than 29 weeks gestation admitted to a tertiary neonatal unit between 1st July 2012 and 30th June 2016 (Infants were divided into two groups based on gestation [23–25 + 6 weeks GA; 26–28 + 6 weeks GA] and birth weight [BW < 1000 g; BW ≥1000 g] for the purpose of multivariate analysis). Total intravenous fluids were commenced at 60 ml/kg/day at birth comprising AA/Dextrose solution aiming to deliver 2 g/kg/day of protein. AA/Dextrose solution contained 33 g/L of Primene 10% (Baxter Pharmaceuticals Pty Ltd) as the amino acid source and 100 g/L dextrose. LE was commenced at birth. The starting dose was 1 g/kg/day and every 24 h the dose was increased by 1 g/kg/day until a maximum of 3 g/kg/day was reached [14]. Plasma triglycerides were measured 24 h after commencement of lipids and then 24 hourly until 3 g/kg/day was reached. If plasma triglycerides were > 2.8 mmol/L, the dose was reduced by 1 g/kg/day. There was a general policy of running a minimum of 0.5 g/kg/day of lipids irrespective of plasma triglycerides to prevent essential fatty acid deficiency, but clinical teams had the choice of ceasing lipids altogether if HT was considered severe. Once 3 g/kg/day of lipid intake was reached triglycerides were measured after 24–48 h and then weekly if they remained on LE infusion. LE infusions were ceased when infants’ enteral feed volume had reached 100–120 ml/kg/day and PN infusions were ceased when enteral feeds had reached 120–140 ml/kg/day.

### Lipid emulsion preparation

LE is delivered in our NICU via amber coloured syringes. Clinoleic 20% (80% Olive oil and 20% Soybean oil) was used as the LE until October 2015. SMOF lipid (30% soybean oil, 30% MCTs, 25% olive oil, and 15% fish oil) was used in the subsequent period. LE syringes are prepared and supplied by the pharmaceutical company (Baxter Pharmaceuticals Pty Ltd). The composition of the LEs are as follows;

#### Clinoleic – 50 mL syringe contains

Clinoleic 20% 36 ml

Vitalipid-N 10% 11.2 ml

Soluvit –N reconstituted in sterile water 2.8 ml.

#### SMOFLipid – 45 mL syringe contains

SMOFlipid 32.5 mL

Vitalipid-N 10% 10 mL

Soluvit-N 2.5 mL

Vitalipid-N™ (Fresenius Kabi Australia Pty Ltd, Sydney, Australia) is a fat soluble vitamin mixture and Soluvit-N™ (Fresenius Kabi Australia Pty Ltd, Sydney, Australia) is a water soluble preparation. LE is infused continuously. LE syringe and administration sets were changed every 48 h.

### Plasma TG concentrations

All TG measurements performed over the first 10 days of life were recorded. HT was defined as plasma TG > 2.8 mmol/L [1]. The clearance of LE is suggested to be saturated at concentrations above 4.5 mmol/L and this was therefore taken as cut off value for severe HT [15]. A minimum of 3 or more TG levels within first 10 days was taken as evidence of compliance to protocol.

### Definitions

Perinatal characteristics and outcomes were sourced from the local Neonatal Intensive Care Units (NICUS) Data collection. Data is collected as part of an ongoing state-wide, prospective data collection from all the neonatal intensive care units for the purpose of quality improvement using consistent definitions. Definitions can be found in the Appendix.

### Statistical analysis

Statistical analyses were performed using IBM SPSS Statistics version 23.0. Categorical outcomes are presented as percentages with odds ratio (OR) and 95% confidence intervals (CI) where appropriate. Continuous variables were compared using the Student t-test or Mann-Whitney U-test. Regression analyses were performed (covariates were included in the model if *p* < 0.1 from the univariate analysis). The South Easter Sydney Local Health District Northern Sector Ethics Committee approved the study.

## Results

**Fig. 1:**
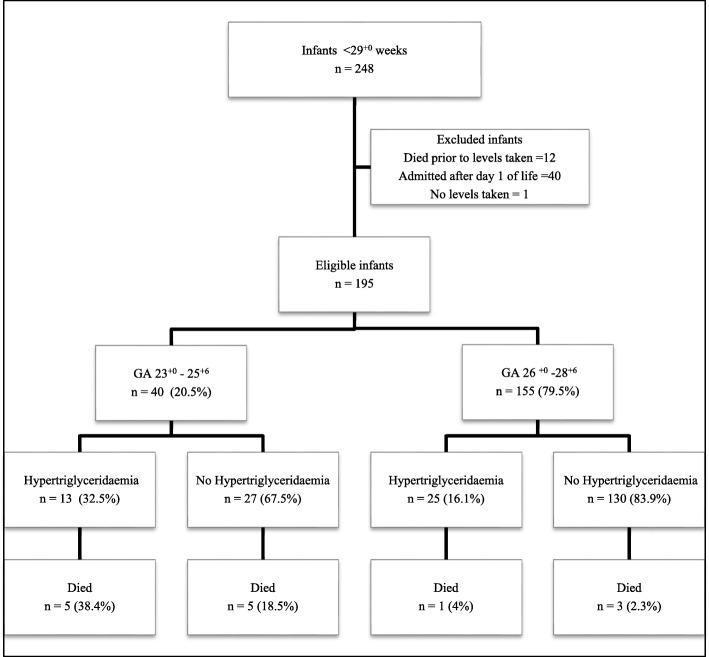
Study population

During the study period, 248 infants born < 29 weeks gestation at birth were admitted. Fifty two were excluded: 12 due to death occurring prior to commencing parenteral nutrition or prior to any levels being taken. Forty infants were admitted after the first day of life. The remaining 196 infants were included in the study (Fig. 1).

### Compliance to TG monitoring protocol

One hundred and seventy four (89%) infants had 3 or more TG measurements and 191 (97%) had at least two TG measurements. Four (2%) infants had only one TG measurement despite receiving LE for at least 3 days. One (0.5%) infant received LE but no TG measurements were done. This infant was not included in the analysis.

### HT group vs Normal TG group

Among 195 infants in whom TG measurements were performed, 38 (19.5%) developed HT, 10 (5.1%) had more than one episode of HT and 11 (5.6%) developed severe HT > 4.5 mmol/L [4 (10.0%) in 23–25^+ 6^ weeks GA and 7 (4.5%) in 26–28^+ 6^ weeks GA]. In all cases of severe HT, the LE was ceased and TG levels returned to normal. There were no overt signs of fat overload directly attributable to LE in these infants, however, two infants developed transient mild thrombocytopenia and one infant developed transient pancytopenia, coinciding with the documented severe HT. There were no episodes of liver dysfunction or cholestasis associated with severe HT. The number of infants who developed HT at 1 g/kg/day, 2 g/kg/day and 3 g/kg/day were 3 (1.5%), 7 (3.6%) and 28 (14.4%) respectively. HT was most commonly observed immediately after an increase in infusion rate.

Pertinent factors in relation to HT are shown in Table 1. The results showed a significantly higher incidence of HT in the 23–25^+ 6^ weeks GA group (32.5% vs 16.1%, p 0.028), with birth weight < 1000 g (29.4% vs 3.9%, *p* < 0.001) and if the infant was SGA (66.7% vs 15.6%, p < 0.001). With respect to severe HT, there was a significantly higher incidence in SGA infants (20.0% vs 4.4%, p 0.042). In the univariate analysis, mortality was significantly higher in HT group (15.8% vs 5.1%, p 0.033). The HT group was more likely to develop severe ROP (31.6% vs 10.2%, p 0.002).

**Table 1:** Maternal and neonatal characteristics and outcomes. Numbers (%) are shown unless otherwise stated

	Normal TG *N* = 157 (80.5)	HT Group *N* = 38 (19.5)	HT vs normal TG *P* value or OR (95% CI; *P* Value)
Multiple Gestation	45 (28.6)	10 (26.3)	0.790
Caesarean delivery	90 (57.3)	28 (73.7)	0.065
Chorioamnionitis	44 (28.0)	7 (18.4)	0.233
Apgar < 7@1 min	102 (65.0)	28 (73.7)	0.315
Apgar < 7@5 min	45 (28.7)	17 (44.7)	0.064
Mean (SD) GA, wk	27 (1)	26 (2)	0.065
Mean (SD) BW, g	1017 (215)	778 (145)	< 0.001
SGA	5 (3.2)	10 (26.3)	< 0.001
Male Gender	87 (55.4)	23 (60.5)	0.577
Early Sepsis	2 (1.3)	1 (2.6)	0.578
Acute Renal Failure	0	0	–
Hepatic Failure	0	0	–
Outcomes			
Mortality	8 (5.1)	6 (15.8)	3.5 (1.13–10.76; 0.041)
Late Sepsis	36 (22.9)	11 (28.9)	1.4 (0.62–3.03; 0.441)
PDA	120 (76.4)	28 (73.7)	0.9 (0.38–1.94; 0.715)
Grade III-IV IVH	10 (6.4)	5 (13.2)	2.2 (0.71–6.95; 0.190)
Stage III-IV ROP	16 (10.2)	12 (31.6)	4.1 (1.73–9.59; 0.002)
NEC	7(4.5)	5 (13.2)	3.2 (0.97–10.86; 0.074)
CLD	64 (40.8)	22 (57.9)	1.9 (0.97–4.09; 0.061)

Numbers (%) are shown unless otherwise stated. GA, gestational age; BW, birthweight; SGA, Small for gestational age; AGA, Appropriate for gestational age

All perinatal factors were analysed in relation to mortality. Apgar scores < 7 at 1 min (64.6% vs 92.9%, p 0.038) and 5 min (29.3% vs 64.3%, p 0.014), gestation 23–25 + 6 weeks (16.6% vs 71.4%, *p* < 0.001), birth weight < 1000 g (59.1% vs 85.7%, p 0.049), grade III-IV IVH (6.1% vs 28.6, p 0.015), and necrotizing enterocolitis (NEC) (5.0% vs 21.4%, p 0.044) were significantly higher in the mortality group. HT was significantly higher in the mortality group (Table 2).

**Table 2:** Hypertriglyceridemia (HT) in relation to mortality. Numbers (%) are shown

	Survived *N* = 181 (92.8)	Died *N* = 14 (7.2)	Died vs survived OR (95% CI; P Value)
HT	32 (17.7)	6 (42.9)	3.5 (1.13–10.76; 0.022)
Severe HT, *N* = 11	8 (4.4)	3 (21.4)	5.9 (1.37–25.4; 0.019)
HT > 1 episode	7 (3.9)	3 (21.4)	6.8 (1.54–29.88; 0.026)

Univariate analysis of mortality and significant morbidities (late sepsis, PDA, grade III-IV IVH, stage III-IV ROP, NEC, CLD) revealed that the HT group had significantly higher mortality and severe ROP in comparison to the normal TG group. A multivariate analysis was performed (acknowledging a limited sample size) for mortality and severe ROP adjusted for all covariates with *p* < 0.1 in the model (gestation 23–25^+ 6^ weeks; HT; SGA). Only lower GA was found to be an independent risk factor for mortality and severe ROP (Tables 3 and 4). Regression analysis was repeated with severe HT as a covariate in place of HT in the model for mortality and the results were similar.

**Table 3:** Regression analysis of risk factors in relation to mortality

	B	S.E.	Wald	Sig.	Exp (B)	95% CI	
						Lower	Upper
23–25^+6^ wk. group	2.515	.657	14.658	.000	12.37	3.41	44.83
HT	.677	.674	1.008	.315	1.968	.525	7.38
SGA	−.883	1.020	.749	.387	.414	.056	3.055
Constant	−3.034	1.035	8.590	.048

**Table 4:** Regression analysis of risk factors in relation to severe ROP

	B	S.E.	Wald	Sig.	Exp (B)	95% CI	
						Lower	Upper
23–25^+6^ wk. group	3.494	.815	18.387	.000	32.923	6.666	162.599
HT	.591	.767	.594	.441	1.806	.402	8.119
SGA	−1.785	1.199	2.214	.137	.168	.016	1.762
Constant	−2.631	1.091	5.815	.016	.072		

## Discussion

Over the past decade numerous studies have shown that commencing lipids shortly after birth are safe with numerous potential benefits [5, 16, 17]. These studies regularly monitored TG concentrations and LE intakes were adjusted accordingly. The majority of units in Australia and New Zealand are now commencing early lipids but regular monitoring is only being practised by a small number of units.

The incidence of HT varies among studies largely due to different TG thresholds, variable patient demographics and different infusion rates of LE. Holtrop et al. [16] commenced infants < 1000 g on 0.5 g/kg/day of Intralipid on day 1 of life and increased by 0.5 g/kg/day measuring lipids when they were on 1 g/kg/day, 2 g/kg/day and then weekly thereafter. They defined HT as TG levels ≥2.3 mmol/L (200 mg/dl) and noted an incidence of 26.7%. Vlaardingerbroek et al. [17], in a randomized controlled trial in infants < 1500 g compared two different AA and lipid doses from birth. Both intervention arms received lipids at a starting dose of 2 g/kg/day on day 1 and then 3 g/kg/day on day 2, whereas the control arm received lipids at a starting dose of 1.4 g/kg/day on day 2 and then 2.8 g/kg/day on day 3. HT (defined as > 3 mmol/L) occurred frequently in all 3 arms with no significant difference (44% in controls vs 27 and 45% in the intervention arms). Drenckpohl et al. [5] commenced the control group on 0.5 g/kg/day and the intervention group on 2 g/kg/day on day one of life and increased by 0.5 g/kg/day until all infants reached 3 g/kg/day. Infants between 750 g and 1500 g were included. They reported HT (TG ≥ 2.27 mmol/L) incidence of 4 and 15% in the control and treatment groups respectively. Neither Vlaardingerbroek [17] nor Drenckpohl [5] published the characteristics of the infants that developed HT or if the infants with HT had increased mortality or morbidity.

Similar to previous studies, our study found that gestation 23–25^+ 6^ weeks GA and lower birth weight were significant risk factors for the development of HT. HT was significantly higher at 26.9% in the < 1000 g group in comparison to only 3 infants (3.9%) in the ≥1000 g group. The high incidence of HT among infants < 1000 g in our study is similar to the findings of Holtrop et al [16]. They found that with each 100 g decrease in BW there was almost twice the odds of an elevated TG. Brans et al. [18], also demonstrated infants < 1000 g are especially at risk of HT. We also found that SGA was a significant risk factor. Despite small numbers, there was also a significantly higher incidence of severe HT in growth restricted infants. Based on these findings, we recommend regular monitoring for HT among infants 23–25^+ 6^ weeks GA (or < 1000 g).

Unlike Holtrop our study found that HT was associated with higher mortality and severe ROP in univariate analysis. This could be due to a lower TG threshold and a smaller sample size used in their study. On multivariate logistic regression analysis our study found that HT was not significant for increased risk of mortality or severe ROP with only lower gestation being an independent risk factor. It is likely that our practice of regular monitoring with prompt titration of lipid intake as per TG thresholds reduced the chance of plasma TG reaching and/or staying at harmfully high level. In cases of severe HT specifically, TG levels returned to normal 24 h after ceasing LE. This may have mitigated any true adverse effect of sustained HT on clinical outcomes in our study. A well-designed large clinical trial is required to evaluate the effect of regular TG monitoring and titration of LE intake on clinical outcomes.

Compliance rate over the study period was 89%. Since this policy has been introduced TG monitoring and titration of LE has become accepted practice among nursing and medical staff particularly in infants < 1000 g. Although compliance was high, we initiated further education sessions to the staff to reach the goal of 100% compliance. LE appears to be well tolerated in infants ≥1000 g. Given the low occurrence in this group and that brief periods of HT appears safe, it seems reasonable to measure TG levels when the infant reaches 3 g/kg/day and then weekly thereafter in infants ≥1000 g. Following review of our guideline, a further audit to check compliance would be completed.

### Limitations

This is a retrospective study and carries an inherent selection and reporting bias. Although, there was a good compliance with the protocol with 89% having TGs monitored at least 3 times in the first 10 days, we did not collect the actual LE intakes received in the first 10 days of life. This would have confirmed how well LE intakes were adjusted in relation to TG concentrations. The fact that high TG concentrations were short-lived indicates good adherence to the unit’s guidelines.

Until October 2015, Clinoleic 20% was used as the lipid emulsions (173 infants) and SMOF lipid (22 infants) for the remaining period. The incidence of both HT and severe HT was not different between the two cohorts. A recent RCT performed by Deshpande et al. also showed similar tolerance of the two preparations in preterm infants [19].

## Conclusion

HT is common in extreme preterm infants, particularly among infants 23–25^+ 6^ weeks GA. Regular monitoring and prompt titration of LE resulted in only short periods of HT potentially avoiding any harmful outcomes. With a lack of evidence that prolonged or severe HT is safe, regular monitoring is recommended especially among extreme preterm infants 23–25^+ 6^ weeks GA (or < 1000 g). Larger studies are needed to clarify the impact of HT on morbidity and mortality.

## Appendix

Definitions: Gestational age was based on best obstetric assessment, using information on ultrasound measures and the date of the last menstrual period. Small for gestational age (SGA): Birthweight percentiles <10th percentiles based on Australian birthweight percentiles by gestational age [20]. Hospital survival: Survival to first discharge home. Intraventricular haemorrhage (IVH): The worst grade of IVH using Papile Classification on ultrasound imaging [21]. Retinopathy of prematurity (ROP): The worst stage as described by the Committee for Classification of Retinopathy of Prematurity [22]. Chronic lung disease (CLD): Any respiratory support at 36 weeks corrected gestational age. Early-onset sepsis: Clinical picture consistent with sepsis within the first 48 h of life and a positive culture of blood and/or cerebrospinal fluid. Late-onset sepsis: Clinical picture consistent with sepsis after the first 48 h of life and a positive culture of blood and/or cerebrospinal fluid [23]. Necrotizing enterocolitis (NEC): defined as per Bell’s criteria [24].

## Abbreviations

ANZNN: Australian and New Zealand Neonatal Network
BW: Birth weight
CI: Confidence interval
CLD: Chronic lung disease
ESPGHAN: European Society for Paediatric Gastroenterology Hepatology and Nutrition
GA: Gestational age
HT: Hypertriglyceridaemia
IUGR: Intrauterine growth restriction
IVH: Intraventricular haemorrhage
NEC: Necrotizing enterocolitis
NICU: Neonatal Intensive Care Unit
NSW: New South Wales
PDA: Patent ductus arteriosus
PN: Parenteral nutrition
ROP: Retinopathy of prematurity
SGA: Small for gestational age
TG: Triglycerides
